# Genomic properties and clinical outcomes associated with tertiary lymphoid structures in patients with breast cancer

**DOI:** 10.1038/s41598-023-40042-7

**Published:** 2023-08-19

**Authors:** Liye Wang, Shuai Gong, Lina Pang, Shengli Zhang, Xiaoke Zhang, Wei He

**Affiliations:** Department of Oncology, the First Affiliated Hospital of Zhengzhou University, No.1 Eastern Jianshe Road, Zhengzhou, 450052 Henan People’s Republic of China

**Keywords:** Breast cancer, Prognostic markers

## Abstract

Tertiary lymphoid structures (TLSs) play a crucial role in determining prognosis and response to immunotherapy in several solid malignancies. Nevertheless, the effect of TLS-associated gene signature based on The Cancer Genome Atlas (TCGA) cohort in patients with breast cancer (BRCA) remains controversial. Based on TCGA-BRCA dataset (n = 866), 9-gene was identified to construct an TLS signature and further analyzed its prognostic value. Then, we explored the relationship of this TLS signature with molecular subtype, immune microenvironment, tumor mutational burden (TMB). High-TLS signature patients had a better overall survival (OS) than low-TLS signature patients, consistent with the results in the METABRIC cohort. Multivariate analysis revealed that TLS signature remained an independent prognostic indicator for OS. In addition, we established a nomogram with the integration of TLS signature and other independent variables to predict individual risk of death. The comprehensive results showed that patients with high TLS signature benefit from immunotherapy; the signature was also correlated with inhibition of cell proliferation pathways, low *TP53* mutation rate, high infiltration of B cells, CD8 + T cells, CD4 + T cells, and M1 macrophages. Therefore, TLS signature is a promising biomarker to distinguish the prognosis and immune microenvironment in BRCA.

## Introduction

Breast cancer (BRCA) has surpassed lung cancer as the most commonly diagnosed cancer worldwide, and is the main cause of tumor-associated mortality in women^1,2^. BRCA is characterized by a high degree of heterogeneity, with divergent histological subtypes and biological characteristics based on hormone receptor (HR) and human epidermal growth factor receptor 2 (HER2) expression^3^, thus leading to distinct clinical behaviors and treatment sensitivity profiles^3^. Important studies have found that tumor-infiltrating immune cells and high expression of immune checkpoints by tumor or immune cells, which are key components of the adaptive immune system with a crucial impact on the prognosis and immunotherapy of BRCA^4,5^. However, the interaction between tumor cells and the tumor microenvironment is a complex and evolving process. Therefore, it is necessary to identify more effective biomarkers for improving precision immunotherapy in BRCA patients.

Tertiary lymphoid structures (TLSs) have very similar structure and development with lymph nodes, also known as tertiary lymphoid organs (TLO) and ectopic lymphoid structures (ELS), which are ectopic lymphocytes aggregates in nonlymphoid tissues under conditions of chronic inflammation and tumors^6^. Mature TLSs are composed of B-cell-enriched zones and T-cell-enriched regions, which contain germinal B-cells centers and dendritic cells surrounded by a rim of T-cells, high endothelial venules (HEVs), as well as lymphatic vessels^7^. Recent evidence revealed that TLSs play a crucial role in determining prognosis and response to immunotherapy^7^. Utilizing transcriptomic analyses to determine TLS-associated gene signatures, which could provide a more global assessment of immune pathway-related signaling and tumor-related immune cell characteristics. Cabrita et al.^8^ identified a unique 9-gene (*CD79B, CD1D, CCR6, LAT, SKAP1, CETP, EIF1AY, RBP5, PTGDS*) expression signature based on differential gene expression between different cases of melanoma, which could predict prognosis and response to immunotherapy^8^. Feng et al.^9^ subsequently interrogated the 9-gene across transcriptomic of 515 lung adenocarcinoma (LUAD) patients in The Cancer Genome Atlas (TCGA) cohort to examine the TLS signature. They found that the 9-gene accurately predicted the presence of TLS and showed TLS signature was an independent positive prognostic factor for LUAD patients. However, little was known about the effect of the same 9-gene signature on the BRCA microenvironment.

Hence, with the publication of new studies regarding TLS at the gene level, further evaluation of the role of abovementioned TLS signature in BRCA is necessary. This study re-used the 9-gene list, but performed exclusive methodology to provide further details on its value for the clinicians and scientists. The aim of this study was to explore the relationship of this 9-gene TLS signature with molecular subtype, immune microenvironment, tumor mutational burden (TMB), and patient outcome and predict response to immunotherapy in BRCA samples by using high-dimensional datasets in the TCGA. Furthermore, the predictive effect of this TLS signature based on the TCGA-BRCA cohort was further validated and supplemented our results using the METABRIC (Molecular Taxonomy of Breast Cancer International Consortium) dataset.

## Results

### TLS signature in breast cancer

We first investigated the expression of 9-gene in the TLS signature both in the BRCA samples and the corresponding normal samples. Compared to normal tissues, significantly higher expression for *SKAP1, LAT* and lower expression for *CD1D, CD79B, CETP, PTGDS* was found in tumor tissues in the TCGA-BRCA cohort (Fig. S1). Then, the prognostic value of all 9-gene was explored both in the TCGA-BRCA and METABRIC datasets. X-tile was used to determine optimal cut-off values for high and low expression regarding all 9-gene for OS. As shown in Figures S2 and S3, the patients with gene high expression had better OS than patients with low expression, while the *EIF1AY* gene was not connected with OS in either dataset (TCGA-BRCA or METABRIC) and the *LAT* gene was not statistically significant in the METABRIC dataset. According to the expression threshold, BRCA patients were divided into high signature group and low TLS signature group. Tables S1 and S2 showed the characteristics of the patients in the TCGA-BRCA cohort (n = 866) and the METABRIC cohort (n = 1399). Next, we further observed that the TLS signature expression was upregulated in the estrogen receptor (ER)-negative group, progesterone receptor (PR)-negative group, HER2-negative group, and triple-negative breast cancer (TNBC) subtype in the METABRIC dataset, while the result showed no statistically significant in HER2 group (Fig. S4A–D). Meanwhile, we also found significant differences in the expression of TLS signature among different molecular subtypes (Fig. S4E).

### Prognostic value of TLS signature

**Figure 1:**
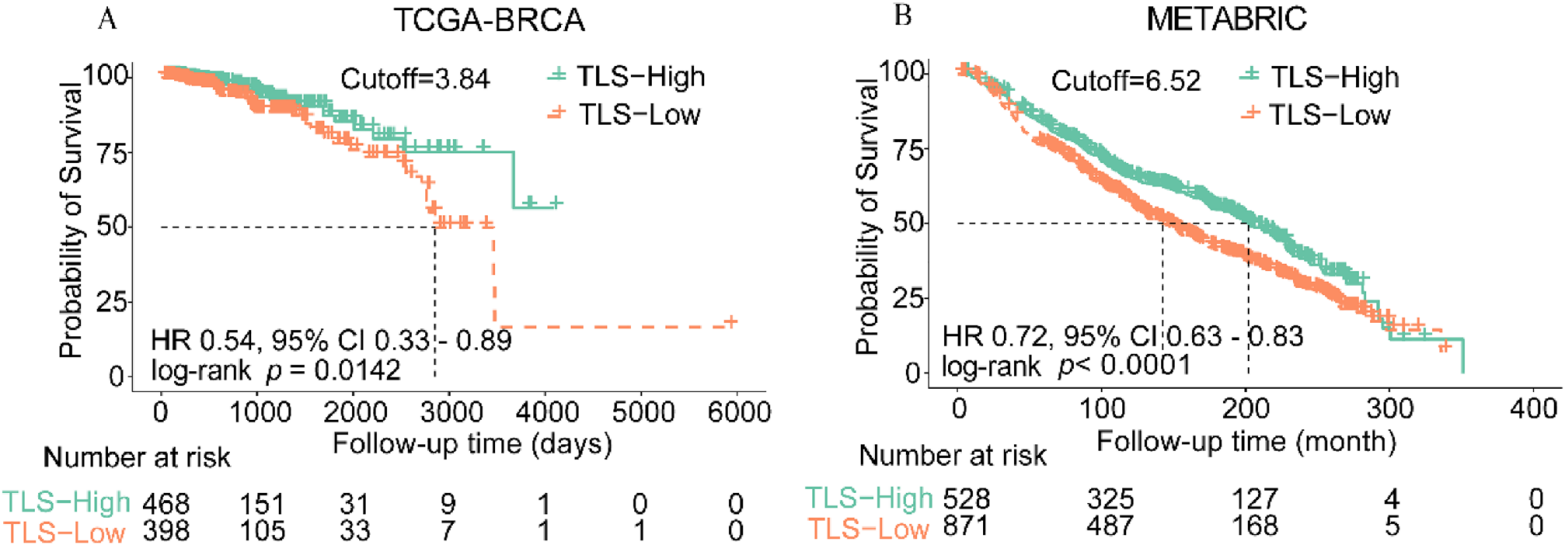
Kaplan–Meier curves for overall survival according to TLS signature expression. Kaplan–Meier curves comparing OS in patients with different TLS signature (*CD79B, CD1D, CCR6, LAT, SKAP1, CETP, EIF1AY, RBP5, PTGDS*) subgroups in the TCGA-BRCA cohort (**A**) and METABRIC validation cohort (**B**).

We aimed to explore the prognostic value of TLS signature in the TCGA-BRCA cohort. X-tile software was used to generate the optimal cut-off value for the TLS signature, we assigned 398 (46%) patients into the low-TLS signature group and 468 (54%) patients into the high-TLS signature group. Patients with high-TLS signature had longer OS (HR 0.54, 95%CI from 0.33 to 0.89, *p* = 0.0142) compared with patients with low-TLS signature (Fig. 1A). In the METABRIC validation cohort of 1399 BRCA patients, TLS signature was also evaluated. With the same method, we assigned 871 (62.3%) patients into the low-TLS signature group and 528 (37.7%) patients into the high-TLS signature group. The patients with high TLS signature also had longer OS (HR 0.72, 95%CI from 0.63 to 0.83, *p* < 0.0001) than those with low-TLS signature (Fig. 1B).

**Figure 2:**
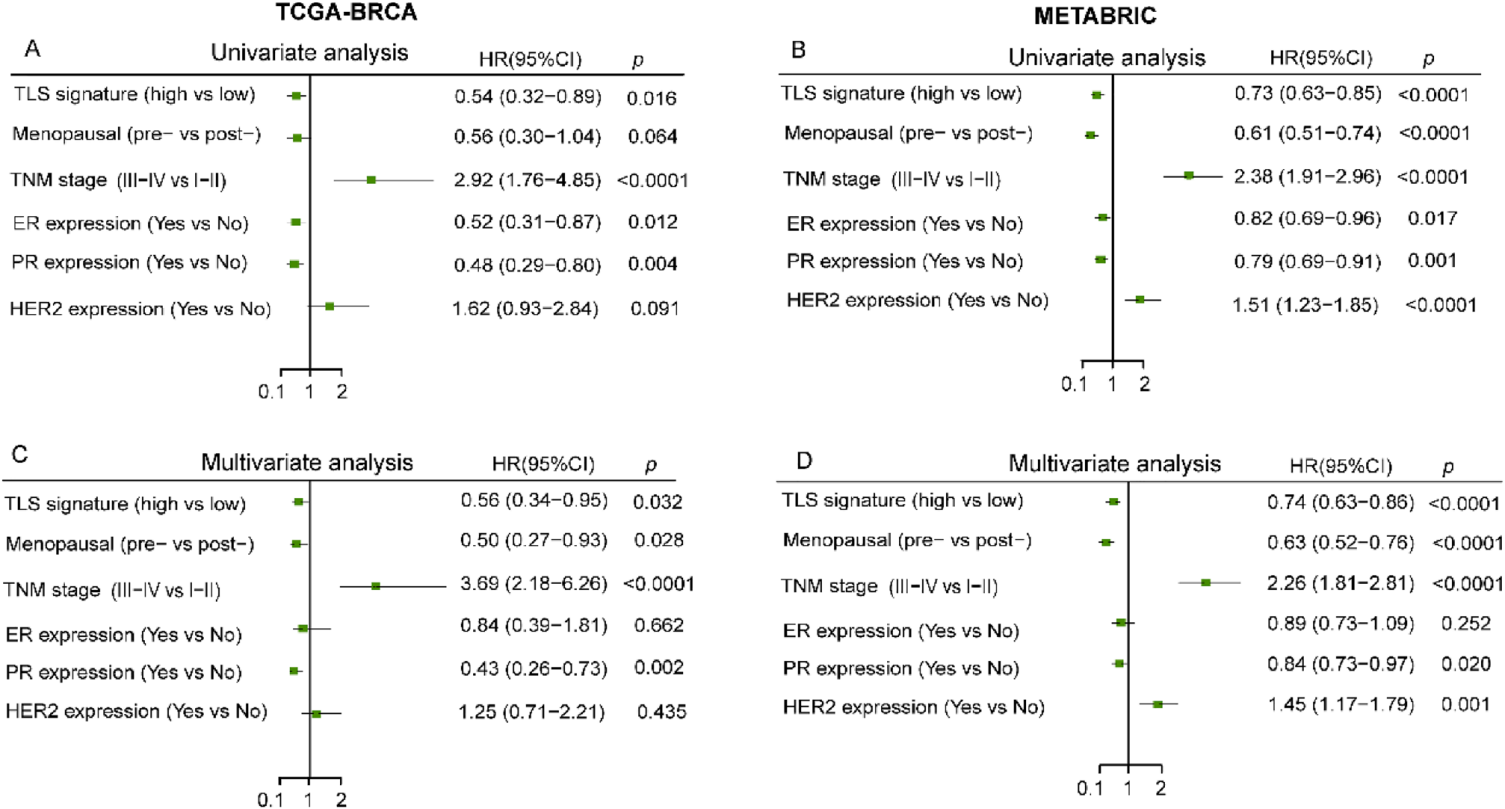
Univariate and multivariate analysis of factors associated with OS. Plots show univariate (**A**) and multivariate (**C**) analysis in the TCGA-BRCA cohort; univariate (**B**) and multivariate (**D**) analysis in the METABRIC validation cohort.

In the TCGA-BRCA cohort, univariate analysis showed that TLS signature, TNM stage, ER and PR expression were significantly associated with OS (Fig. 2A). Multivariate Cox regression analysis showed that the TLS signature remained significant for OS (HR 0.56, 95% CI from 0.34 to 0.95, *p* = 0.032 Fig. 2C). These results were validated by METABRIC cohort (Fig. 2B, D). In addition, the menopausal, TNM stage and PR expression were found statistically significant in both TCGA and METABRIC cohort (Fig. 2C, D).

### Development of nomogram with TLS signature

**Figure 3:**
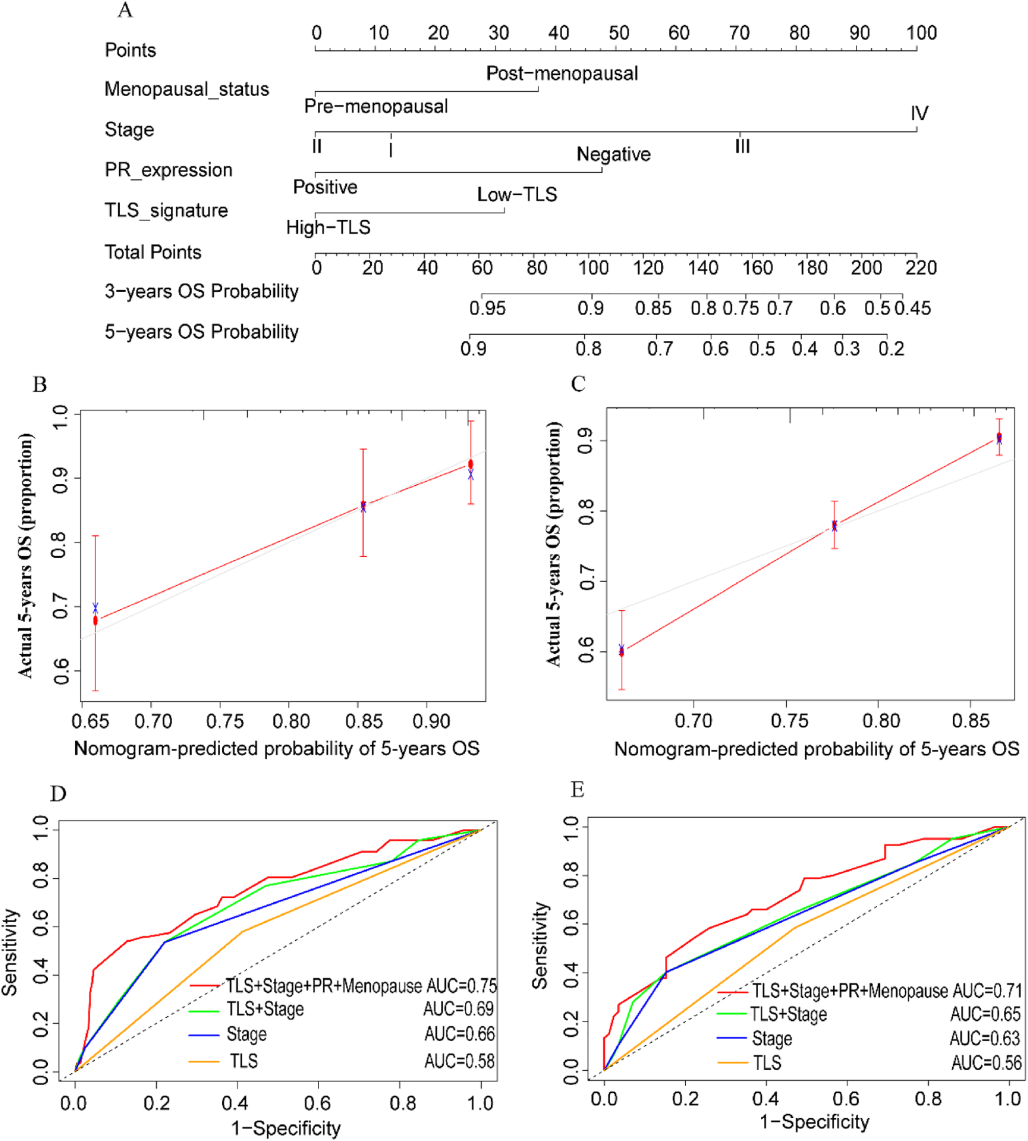
Nomogram and calibration plots for predicting 5-year OS. (**A**) Nomogram including menopausal status, TNM stage, PR expression and TLS signature; the calibration plots predicting 5-year OS in BC patients from the TCGA-BRCA cohort (**B**) and METABRIC validation cohort (**C**); comparisons of the sensitivity and specificity for the prediction of 3-year (**D**) and 5-year (**E**) OS by the combined TLS, TNM stage, PR and menopause model, the TLS and TNM stage model, the TNM stage alone model, and the TLS alone model.

We constructed nomogram combining TLS signature, menopausal status, TNM stage and PR expression to predict the 3-year and 5-year OS of BRCA patients based on the multivariate Cox regression analysis in the TCGA-BRCA cohort (Fig. 3A). The concordance index (C-index) for the nomogram to predict OS was 0.746 (95%CI from 0.703–0.789). Subsequently, the calibration plot of nomogram for the probability of 5-year OS showed good agreement between the prediction by nomogram and actual observation for nomogram in the TCGA-BRCA cohort (Fig. 3B). This was the same as in the METABRIC validation cohort (Fig. 3C). Meanwhile, we used ROC analysis to compare the sensitivity and specificity of the prognostic model with the TLS signature combined with TNM stage, or the separate model for TNM stage or TLS signature. Combination of the TLS signature, menopausal status, TNM stage and PR showed better prognostic value than the TLS signature and TNM stage (AUC: 0.75 vs 0.69), TNM stage alone (AUC: 0.75 vs 0.66), TLS signature alone (AUC: 0.75 vs 0.58) for 3 years OS and 5 years OS (AUC: 0.71 vs 0.65 vs 0.63 vs 0.56) in the TCGA cohort (Fig. 3D, E).

### Molecular characteristics of different TLS signature subgroups

To further explore TLS signature related biological processes in BRCA, a total of 2987 differentially expressed genes (DEGs) and 2849 DEGs (see method), sieved from the TLS signature high (Fig. S5A) and low (Fig. S5B) groups in the TCGA-BRCA cohort, respectively. Subsequently, GO (Gene Ontology Resource, Fig. S5 C, D) and KEGG^10^ (Kyoto Encyclopedia of Genes and Genomes, Fig. S5E, F) enrichment analyses were performed to investigate potential biological functions of different TLS signature subgroups. Some processes were found in both sets, but their regulation in high-TLS and low-TLS groups were different. Next, we further investigate TLS signature related immune functions in BRCA. By intersecting the above DEGs with the list of immune-related genes obtained from ImmPort, a total of 226 immune-related DEGs and 237 immune-related DEGs were seived from the TLS signature high (Fig. S6A) and low (Fig. S6B) groups, respectively. The functional enrichment analysis of GO and KEGG are shown in Supplementary Fig. S6.

### Relationship between TLS signature and driver gene mutations

Next, we explore the relationship between TLS signature and TMB. As a result, the TMB in the low-TLS signature group was significantly higher than that in the high-TLS group (*p* < 0.0001, Fig. S7A). Moreover, we observed TLS signature was slightly negatively correlated with TMB in the TCGA-BRCA (Fig. S7B) and METABRIC (Fig. S7C) cohorts.

**Figure 4:**
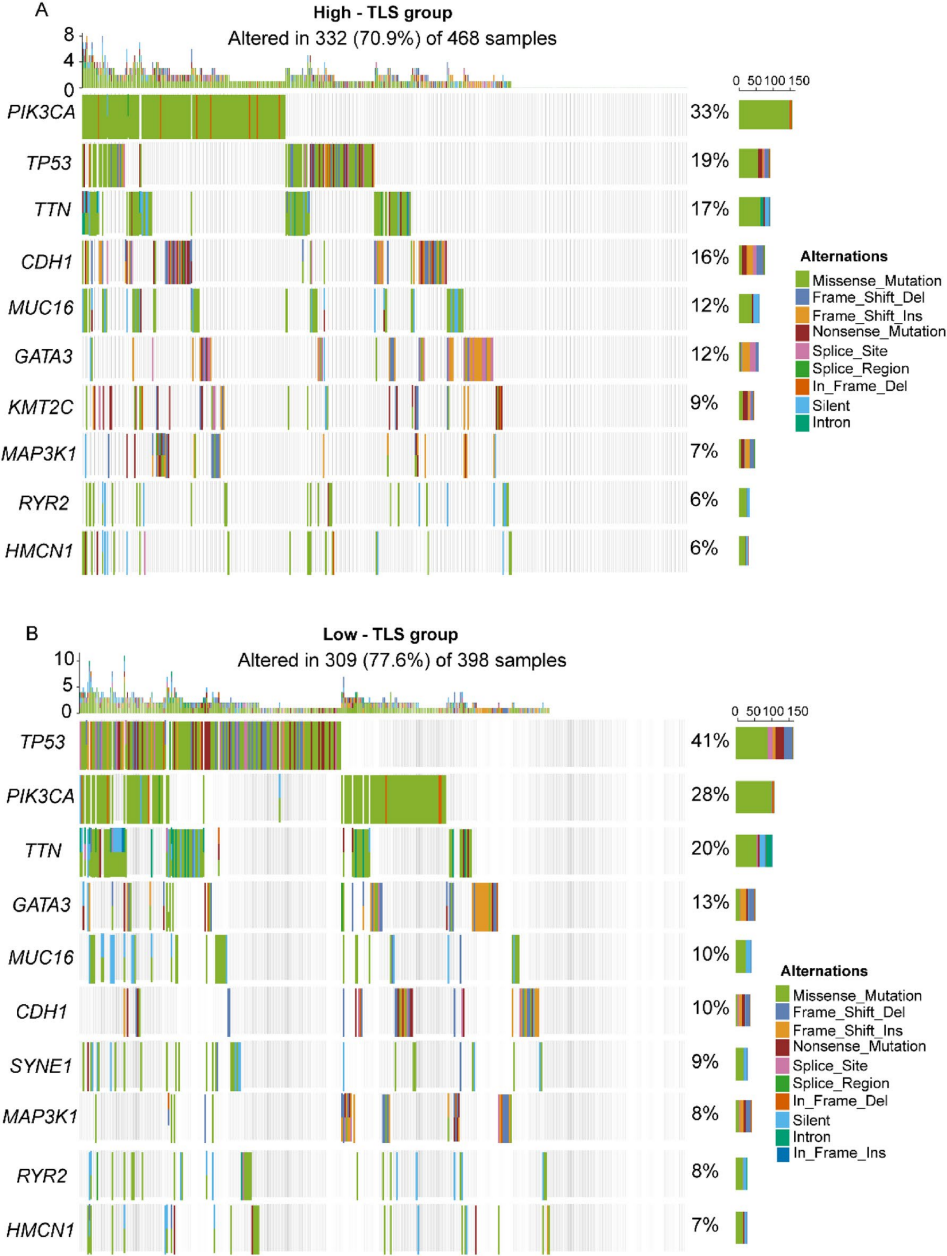
Significantly mutated genes in different TLS signature subgroups. Plots show the mutated genes in the low TLS signature (**A**) and high TLS signature (**B**) groups. Mutated genes (rows, top 10) are ordered by mutation rate; samples (columns) are arranged to emphasize mutual exclusivity among mutations. The right shows mutation percentage, and the top shows the overall number of mutations. The color-coding indicates the mutation type. The heatmap was plotted using the complexHetmap package of R (version 2.14.0, URL: https://github.com/jokergoo/ComplexHeatmap).

Next, we analyzed driver gene mutations to gain further biological insight into the immunological nature of the TLS signature subgroups. We found significantly higher mutation frequency in the low TLS signature subgroup than in the high TLS signature subgroup (*p* < 0.0001). Missense variations were the most common mutation type, followed by frameshift and nonsense variations. Subsequently, we identified top 10 genes bearing the highest mutation rates in different TLS signature subgroups (Fig. 4A, B). The mutation rates of *PIK3CA, TP53, TTN, CDH1, MUC16, and GATA3* were higher than 10% in both groups. The mutation of the *KMT2C* was more common in the high-TLS signature subgroup, while the mutation of *SYNE1* was more common in the low-TLS signature subgroup.

### Immune characteristics of different TLS signature subgroups

**Figure 5:**
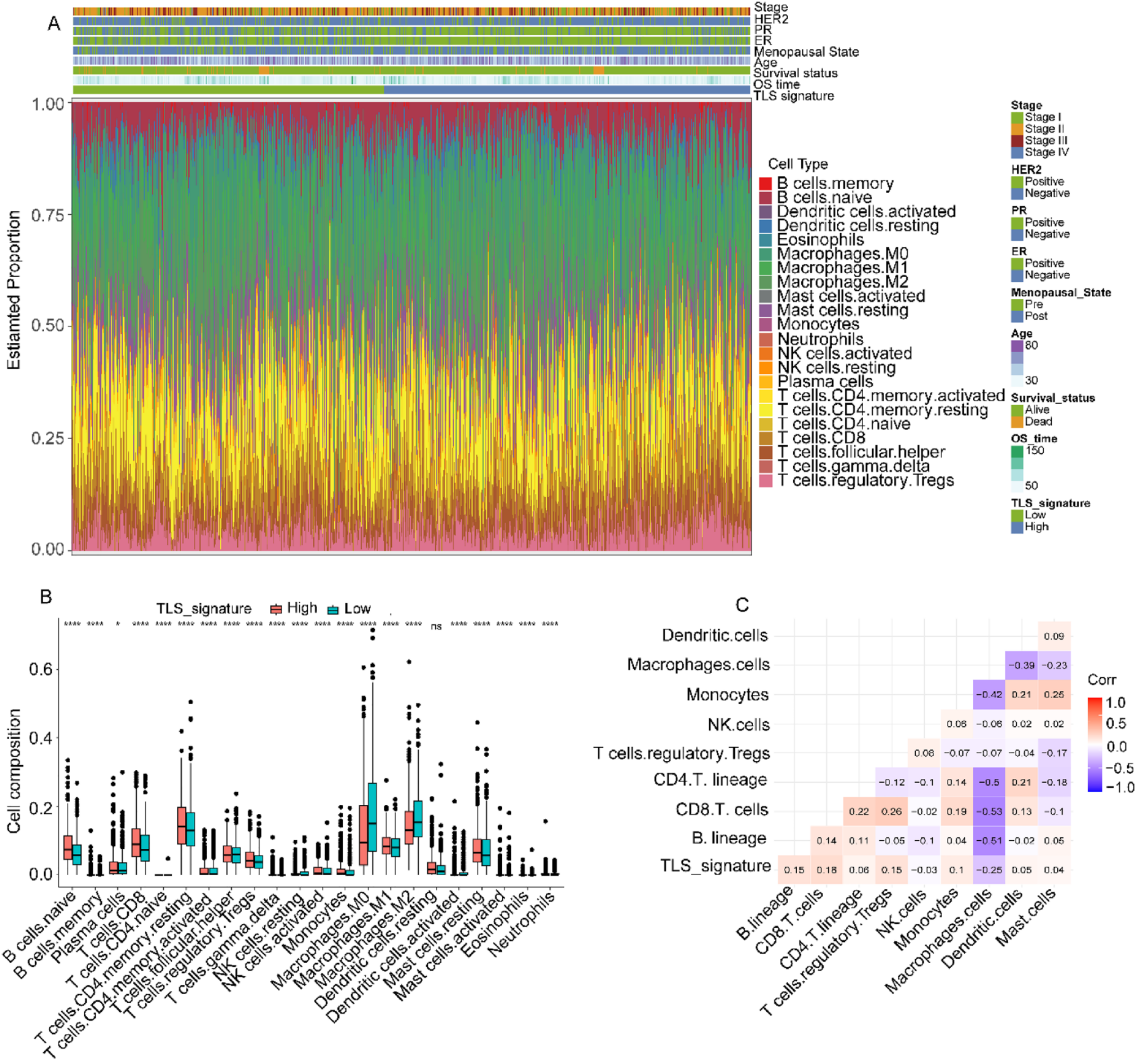
The landscape of the tumor microenvironment (TME) in breast cancer (BC) and the characteristics of different TLS signature subgroups. (**A**) The TLS signature grouping and proportions of TME cells for BC patients in the TCGA-BRCA cohort. Age, Menopausal state, ER, PR, HER2, Stage, OS time, and survival status are shown as patient annotations. The proportions of TME cells in different TLS signature subgroups (**B**). The thick lines represent the median value. The bottom and top of the boxes are the 25th and 75th percentiles (interquartile range), respectively. The scattered dots represent the corresponding subgroups in the graph. Significant statistical differences between the two subgroups were assessed using the Mann–Whitney test (ns: not significant, *p < 0.05, **** *p* < 0.0001). Correlation analysis between TLS signature and immune cells (C). The heatmap was plotted using the pheatmap package of R (version 1.0.12, URL: http://bioconductor.org/packages/release/bioc/vignettes/InteractiveComplexHeatmap/inst/doc/interactivate_indirect.html).

We further analyzed the immune landscape characteristics of different TLS signature subgroups, including the clinicopathological characteristics (showed in Fig. 5A). To analyze the composition of immune cells, we compared the distribution of immune cells in different TLS signature subgroups. We found that naïve B cells, memory B cells, plasma cells, CD8^+^ T cells, resting memory CD4^+^ T cells, activated memory CD4^+^ T cells, regulatory T cells (Tregs), activated NK cells, monocytes and M1 macrophages were more abundant in the high TLS signature subgroup, while follicular helper T cells, M0 and M2 macrophages were more abundant in the low TLS signature subgroup (Fig. 5B). Furthermore, we observed that TLS signature was correlated with immune cell population of B lineage cells, CD8^+^ T cells, CD4^+^ T cells and monocytes, but not with NK cells and macrophages cells (Fig. 5C).

**Figure 6:**
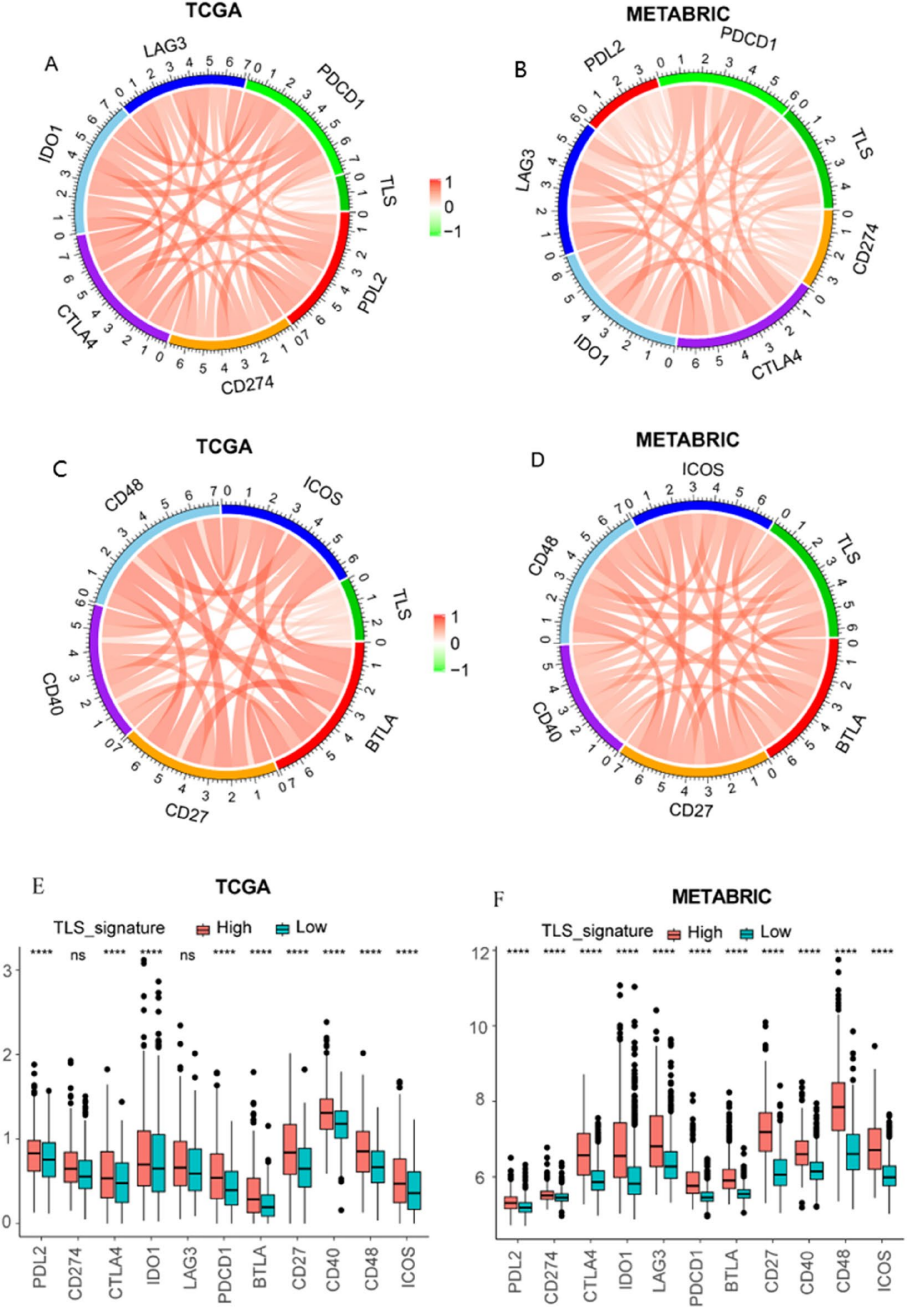
TLS signature is correlated with immune checkpoint members in TCGA-BRCA (**A**, **C**) and METABRIC databases (**B**, **D**). Comparison of immune-related checkpoint genes between the high- and low-TLS signature groups in TCGA-BRCA (**E**) and METABRIC (**F**) databases.

Next, we analyzed the relationship of TLS signature with other immune checkpoint members to further explore the synergistic role of TLS signature in BRCA-induced immune responses. The detailed R and *p*-values of correlations between TLS signature and other immune checkpoint members were listed in Table S3. We found that the TLS signature was positively correlated with other immune checkpoint members expression (Fig. 6A–D). Interestingly, we also found that the expression of all immune checkpoint members was significantly upregulated in the high-TLS signature subgroup compared with the low-TLS signature subgroup (Fig. 6E–F).

## Discussion

Based on the publicly available large-scale BRCA genome datasets and the corresponding clinical information, we quantified TLS signature in each sample using the previously defined 9-gene signature and comprehensively evaluated its correlation with prognosis and molecular characteristics in BRCA. In the present study, we found that the TLS signature was an important factor in predicting prognosis in BRCA patients, which was validated using METABRIC data. Furthermore, the TLS signature was found to be a powerfully independent predictor of OS. Then, we developed a nomogram to predicted the 3-year and 5-year OS of BRCA patients combining all independent variables TLS signature, menopausal status, TNM stage and PR. Furthermore, we characterized the molecular and immune profile of TLS signature in TCGA-BRCA dataset, and further revealed the possible mechanisms how the level of TLS signature in BRCA influence prognosis. Additionally, the TLS signature was positively associated with several immunotherapy response biomarkers.

BRCA was previously considered a relatively poorly immunogenic tumor compared with other tumor types. Recent evidence has suggested that TLSs have attracted increasing attention as a unique structure of the TME in BRCA^11,12^. Based on H&E (hematoxylin–eosin) slides with one or two markers to evaluate TLS, the density and distribution of TLS is a valuable prognostic biomarker in various types of cancers^13,14^. However, the ability to measure the presence of TLSs using H&E slides is highly subjective, less reproducible, and inter-observer and intra-observer variability. However, RNA-seq could not only identify the presence of TLS and but also is ideal for prognosis analysis. Cabrita et al.^8^ demonstrated that a 9-gene TLS signature mainly represented the B cells and T cells in TLSs, which predicted clinical outcomes in cohorts of patients treated with immune checkpoint blockade. Many studies have presented this 9-gene signature has been used for TLS quantification, conveying significant prognostic and predictive value^9,11^. Here, we also measured the presence of TLS using the same 9-gene TLS signature. Moreover, TLS signature was able to predict the survival of BRCA patients in the TCGA-BRCA cohort, which was validated in the METABRIC external cohort. The results also showed that patients with high-TLS signature were associated with early TNM stage, ER-negative, PR-negative and HER2-negative group.

Currently, TLS has become a clinically useful prognostic marker in a variety of cancers, such as melanoma, lung cancer, colorectal cancer^8,9,15^. Wang et al.^11^ showed that the TLS signature could predict prognosis of BRCA patients, and patients with high TLS signature had longer OS. Our study also showed that BRCA patients with high-TLS signature had significantly longer OS than those with low-TLS signature. Importantly, our results demonstrated that TLS signature was an independent prognostic factor for OS. In addition, a prognostic score model combined the TLS signature, menopausal status, TNM stage and PR was constructed, and had a better prognostic value than the TNM stage and TLS signature, which could be an attractive tool in guiding treatment selection. Therefore, the comprehensive 9-gene signature, representing the TLS-associated gene expression signature, can help to understand the immune state of tumors in individuals. Meanwhile, we further explore the molecular characteristics of different TLS signature subgroups. The results suggested that the high-TLS signature subgroup was characterized by inhibition of cell proliferation and inflammatory pathways. Next, we further investigated TLS signature related immune functions in BRCA. We found that TLS signature related genes were mainly involved in immune-related and inflammatory pathways, including cytokine, chemokine and leukocyte mediated signaling pathways. Therefore, this may lead to the different predictive effects of TLS signature subgroups on survival.

To gain further biological insight into the immunological nature of TLS signature subgroups, we then studied gene mutations of different subgroups. The potential predictors of response to immune checkpoint inhibitors (ICIs) in BRCA, including PD-L1 expression, tumor-infiltrating lymphocytes (TILs), tumor mutational burden (TMB), and several other biomarkers^16^. High TMB is a leading candidate biomarker for identifying patients with cancer who may benefit from ICIs^17,18^. However, McGrail et al.^19^ showed that high TMB fails to as a biomarker for prediction ICIs response in all solid cancer types, such as BRCA, because there was no relationship between CD8^+^ T cell levels and neoantigen burden. Our study revealed that TMB of low-TLS signature group was significantly higher than that in the high group. Moreover, we observed TLS signature was slightly negative correlated with TMB. The largest difference in mutations between two groups was *TP53* mutations, which were more common in low-TLS signature samples than high-TLS signature samples (41% vs. 19%). *TP53* is a tumor suppressor gene which is commonly mutated in various cancers including BRCA^20^. *TP53* mutation is linked with more aggressive disease and poorer patient outcomes in many cancers^21,22^. Therefore, low-TLS signature patients with high *TP53* mutation have a worse outcome than high-TLS signature patients with low *TP53* mutation, in agreement with our survival results.

Recently, tumor-infiltrating immune cells in tumor microenvironment (TME) have received increased attention, which closely have an impact on the development, progression, and prognosis as well as the treatment of BRCA^23,24^. Recent evidences revealed that TLSs are highly correlated with immune cell infiltration^11,25^. The composition of immune cells was different between two TLS signature subgroups. B cells, cytotoxic CD8^+^ T cells, CD4^+^ T cells, Treg cells and M1 macrophages were more enriched in the high-TLS signature subgroup, while follicular helper T cells, M0 and M2 macrophages were more common in the low-TLS signature subgroup. Previous studies have shown that the immunosuppressive subsets like Treg cells, which are also components of TLSs, and the association of immunosuppressive cells with TLSs^8,18^. A substantial body of research has revealed that the associations of TLS with abundant immune subset, including immunosuppressive cells^8,9,11^. Previous studies have revealed that dense infiltration of B cells, cytotoxic CD8^+^ T cells, CD4^+^ T cells and M1 macrophages, indicates a favorable prognosis^26–28^. Conversely, a high density of M2 macrophages have been found to favor tumor growth and is associated with a poor outcome in breast^28,29^. Therefore, low-TLS signature patients have a worse outcome than high-TLS signature patients, in agreement with our survival results.

Additionally, we investigated the associations between the TLS signature subgroups and several immune checkpoint members. We found that high-TLS signature patients had significantly higher expression of immune checkpoint members. The results indicated that high-TLS signature patients were more likely to benefit from immunotherapy^11^. Previous studies have revealed that TLS-rich tumors were related to significantly better survival after treated with immune checkpoint blockade on the basis of the TLS signature^8,9,11^. Meanwhile, recent studies had also emphasized the clinical significance of TLS in predicting response to neoadjuvant immunotherapy in patients with lung cancer and melanoma^7,30^. Based on the above results and discussion, the 9-gene signature may have important implications in immunotherapy against BRCA.

To conclude, our study demonstrated that TLS signature, which was based on the expression of 9-gene signature, could predict risk of individual death of BRCA patients in both the TCGA-BRCA cohort and METABRIC dataset. Moreover, we established a nomogram with the integration of TLS signature and other independent variables that might offer clinicians a useful tool for predicting prognosis of BRCA patients. The presence of TLSs could be a promising predictor of response to immunotherapy in BRCA patients.

## Materials and methods

### Patients and datasets

RNA-seq data (FPKM) of 979 BRCA samples, including 866 cancer samples and 113 para-cancer samples, and their clinicopathological information were downloaded from the TCGA database (https://portal.gdc.cancer.gov/) (access date: December 15, 2022). RNA-seq data (FPKM) of 1399 BRCA samples and their clinicopathological information were downloaded from the METABRIC (Molecular Taxonomy of Breast Cancer International Consortium) dataset^31^ (http://www.cbioportal.org/) (access date: November 30, 2022), used as the validation cohort. The list of immune-related genes (Table S4) were downloaded from the ImmPort (https://www.immport.org/shared/home)^32^.

### TLS signature

9 signature genes (*CD79B, CD1D, CCR6, LAT, SKAP1, CETP, EIF1AY, RBP5, PTGDS*)^8^ expression was extracted from the TCGA-BRCA and METABRIC database. The TLS signature score was calculated as the mean gene expression. X-tile software (version 3.6.1; Yale University, New Haven, CT, USA)^33^ was used to determine the optimal cut-off values for high and low TLS signature based on the associations with patient overall survival (OS). Overall survival (OS) was calculated from the date of initial diagnosis to the date of death or the latest follow-up. Patients with a high expression of TLS signature score (TCGA-BRCA > 3.84, METABRIC > 6.52) were assigned as the high-TLS group, and patients with a low expression of TLS signature score (TCGA-BRCA ≤ 3.84, METABRIC ≤ 6.52) were assigned as the low-TLS group.

### Comprehensive analysis of molecular and immune characteristics in different TLS signature subgroups

In bioinformatics analysis, differential expression analysis (*p*-value < 0.05, |log2FC|> 0.585) was first performed on all genes to analyze the samples with high (n = 468) and low (n = 398) TLS signature using the limma package of R (version 3.54.0). Enrichment analysis to determine the biological information in which the differentially expressed genes (DEGs) are involved was then performed using clusterProfiler package of R. Simple nucleotide variation (SNV) and masked somatic mutation date were downloaded from the TCGA database. In gene mutation analysis, both the quantity and quality were considered in two TLS signature subgroups by using the Maftools package of R (version 2.14.0). Spearman correlation analysis were performed between TLS signature and TMB.

To identify immune characteristics of 866 BRCA samples in the TCGA dataset, their expression data were imported into CIBERSORT (https://cibersort.stanford.edu/) and iterated 1000 times to estimate the relative proportion of 22 types of immune cells. The 22 types of infiltrating immune cells inferred by CIBERSORT include B cells, T cells, natural killer cells, macrophages, dendritic cells, eosinophils, and neutrophils. Then, we compared the relative proportions of 22 types of immune cells and clinicopathological factors between the two TLS signature subgroups. Spearman correlation analysis were performed between TLS signature and immune cells and other immune checkpoint members.

### Statistical analyses

Clinicopathological variables associated with TLS signature were analyzed using the χ2 test or Fisher’s exact test. Differences between groups were assessed using the Mann–Whitney U test or one-way Anova for continuous variables. The Kaplan–Meier method was used to estimate OS and differences were compared using the log-rank test. Multivariate Cox regression analysis with backward selection was performed to test the independent significance of different factors. Multivariate analysis was performed using variables with *p* < 0.1 in the univariate analysis, and only independent prognostic factors were retained in the multivariate model. In addition, we established a prognostic model combining the TLS signature, menopausal status, PR and TNM stage. Moreover, nomograms predicting 3 years or 5 years OS were established. The model performance was evaluated by the accuracy of point estimates of the survival function (calibration). The performance of the nomograms was evaluated using the concordance index (C-index)^34^. In addition, bootstraps with 1000 resamples were applied to internal validation to provide an unbiased estimate of model performance.

Statistical analyses were performed with software programs (SPSS version 26.0 (IBM); R version 4.1.2; GraphPad Prism 8). All statistical tests were two-sided and *p* < 0.05 was considered to have the statistically significant difference.

### Supplementary Information

Supplementary Information 1.

Supplementary Information 2.

Supplementary Information 3.

## Data Availability

Publicly available datasets were analyzed in this study. This data can be found here—TCGA: https://portal.gdc.cancer.gov/; METABRIC: http://www.cbioportal.org.
