# Supplementary material for: Genomic properties and clinical outcomes associated with tertiary lymphoid structures in patients with breast cancer
Source: Sci Rep. 2023 Aug 19;13:13542. doi: 10.1038/s41598-023-40042-7 (PMC10439954; doi:10.1038/s41598-023-40042-7)
Supplement: Supplementary file 2 — Supplementary Information 2. [file 41598_2023_40042_MOESM2_ESM.docx]

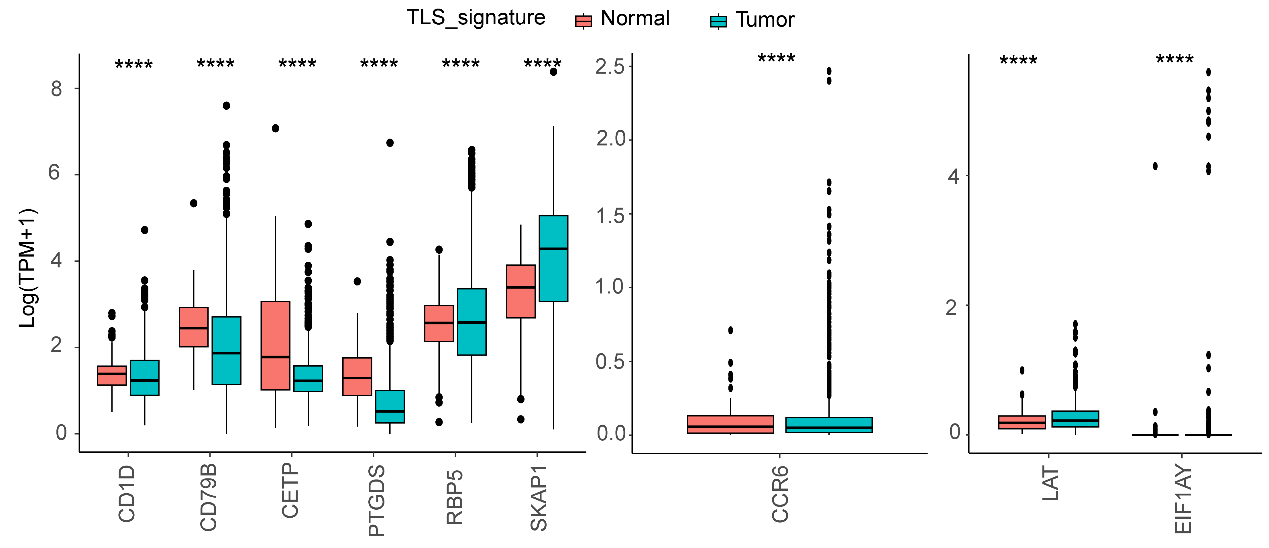


Figure S1 The expression of 9 TLS signature genes in tumor versus normal tissues in the TCGA-BRCA cohort. The scattered dots represent the gene expression of the two groups. The thick lines represent the median value. The bottom and top of the boxes are the 25th and 75th percentiles (interquartile range), respectively. Significant statistical differences between the two groups were assessed using the Wilcoxon test (**** *p* < 0.0001).


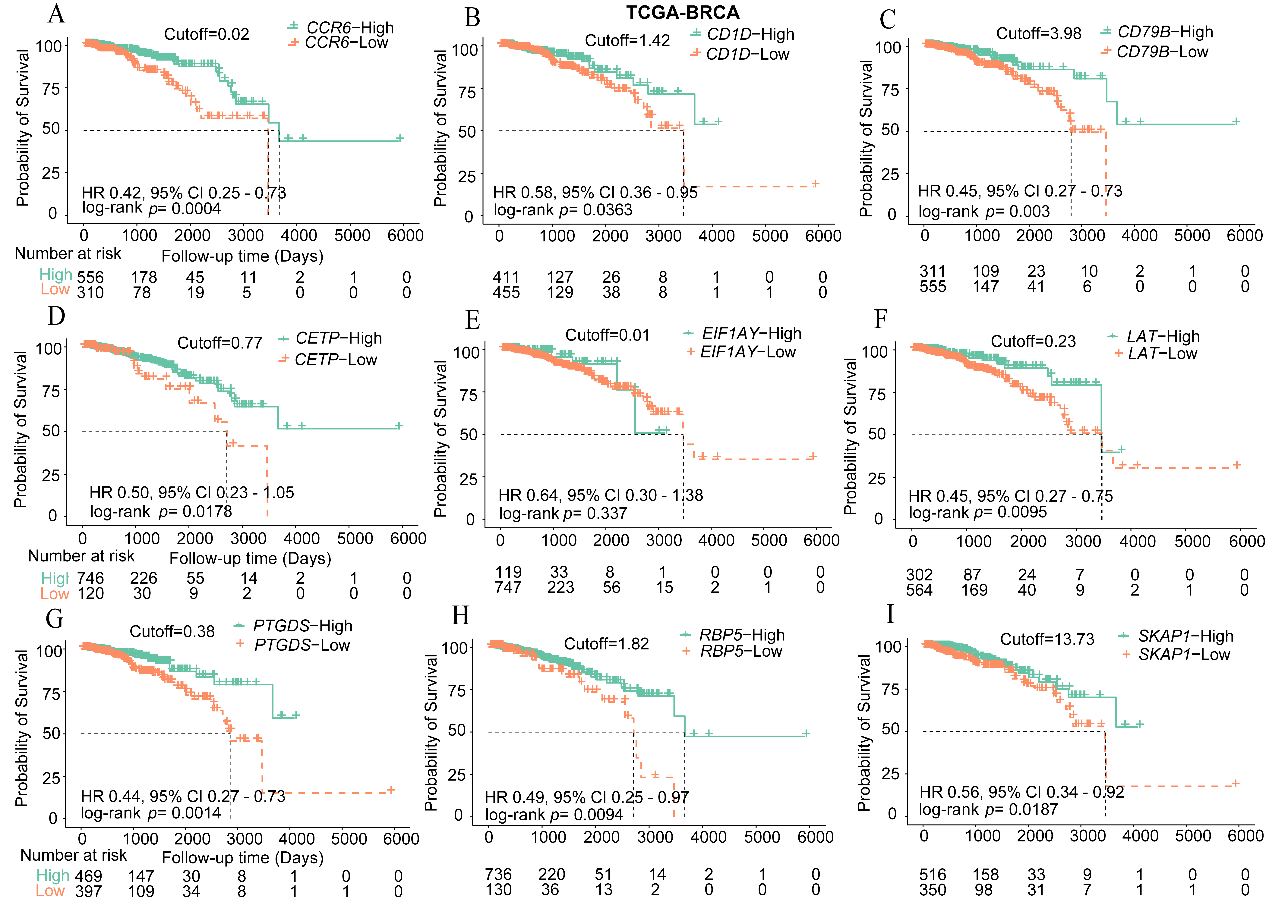


Figure S2 Kaplan-Meier curves for OS according to 9 TLS signature genes expression. Plots show (A) *CCR6*; (B) *CD1D*; (C) *CD79B*; (D) *CETP*; (E) *EIF1AY*; (F) *LAT*; (G) *PTGDS*; (H) *RBP5*; and (I) *SKAP1* in the TCGA-BRCA cohort.


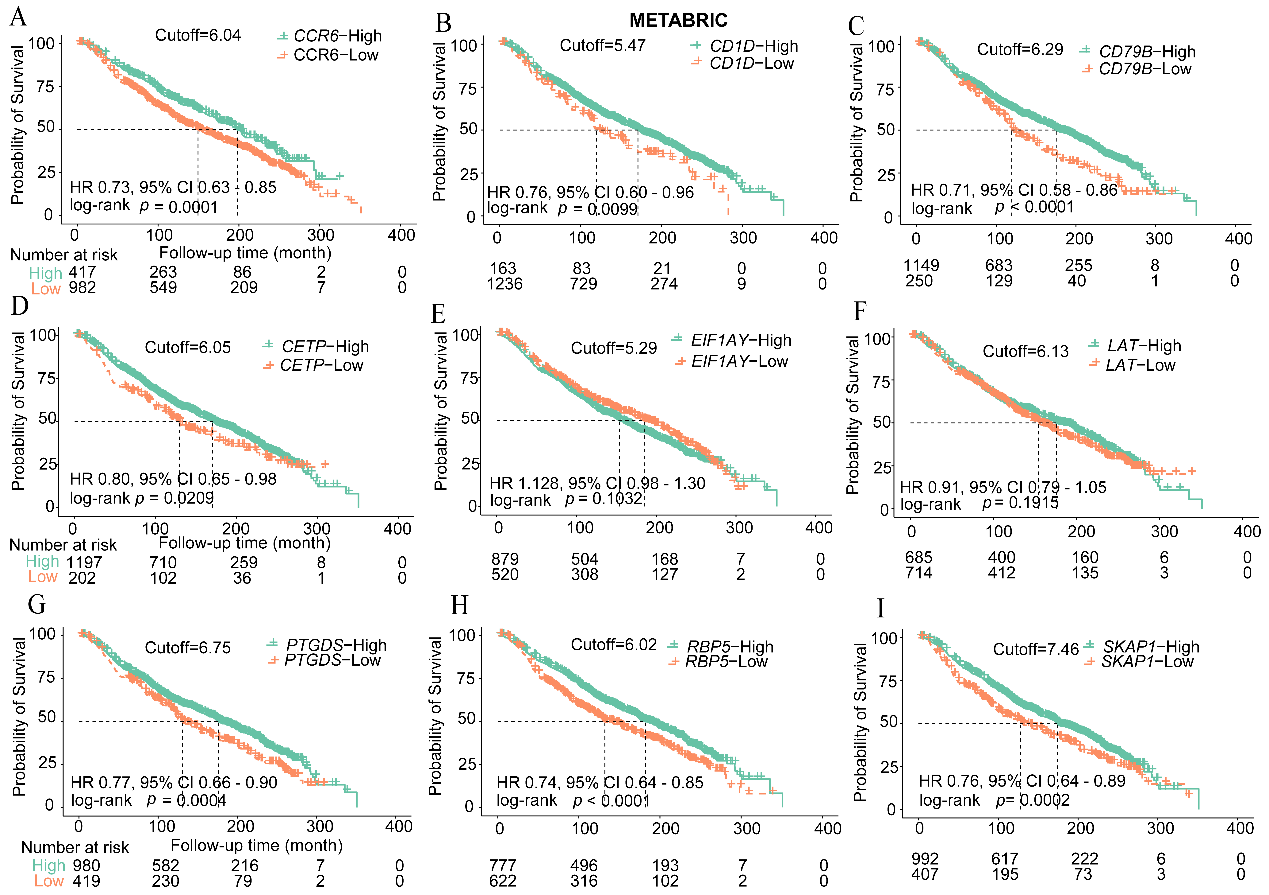


Figure S3 Kaplan-Meier curves for OS according to 9 TLS signature genes expression. Plots show (A) *CCR6*; (B) *CD1D*; (C) *CD79B*; (D) *CETP*; (E) *EIF1AY*; (F) *LAT*; (G) *PTGDS*; (H) *RBP5*; and (I) *SKAP1* in the METABRIC cohort.


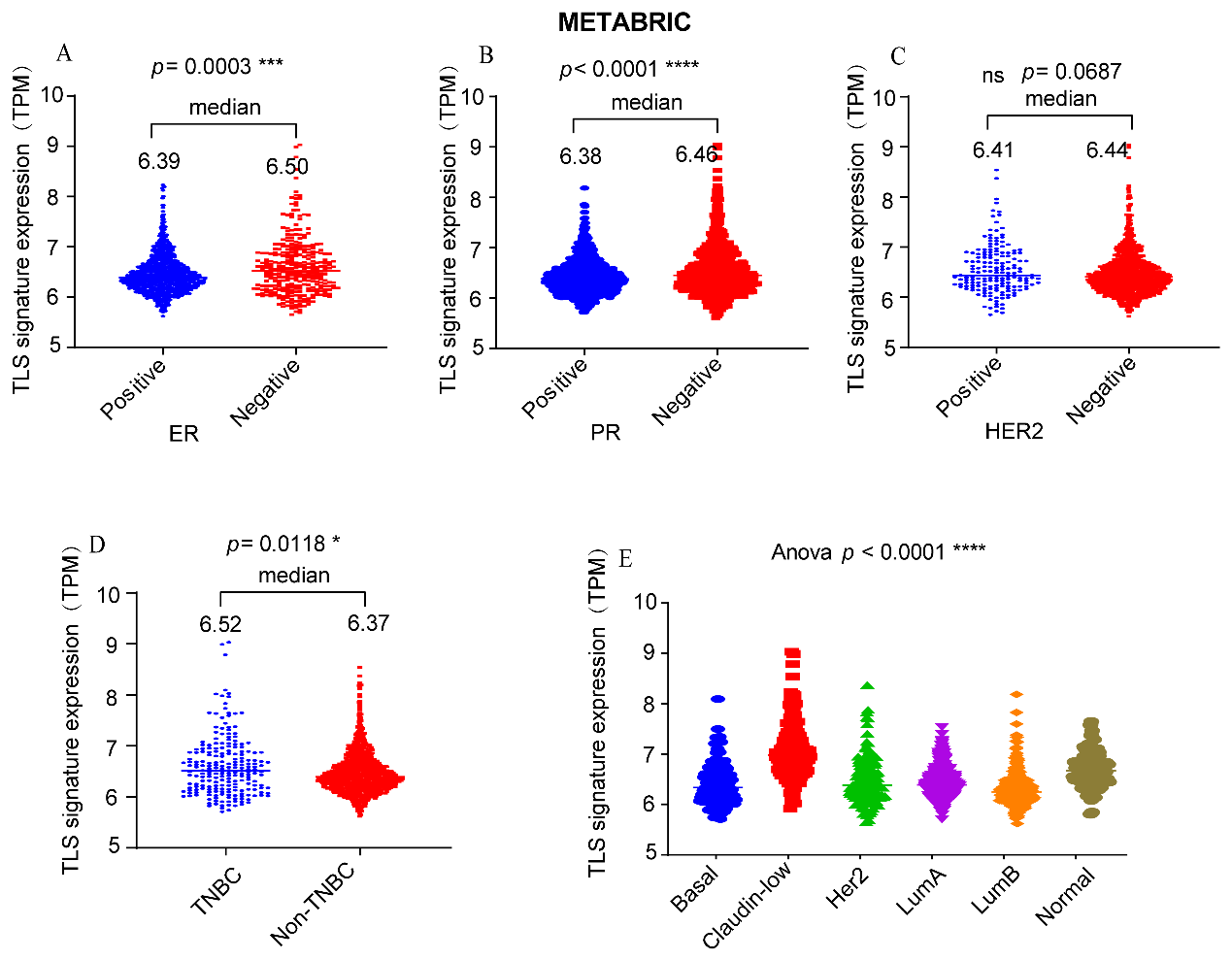


Figure S4 The expression of TLS signature in different ER, PR, and HER2 status (A-C), TNBC and non-TNBC tissues (D), and molecular subtypes (E) in the METABRIC cohort. Significant statistical differences in different groups were assessed using the Wilcoxon test or Anova test (ns: not significant, **p* < 0.05, *** *p* < 0.001, **** *p* < 0.0001). Abbreviations: TLS, tertiary lymphoid structure; ER, estrogen receptor; PR, progesterone receptor; HER2, human epidermal growth factor receptor 2; TNBC, triple negative breast cancer.


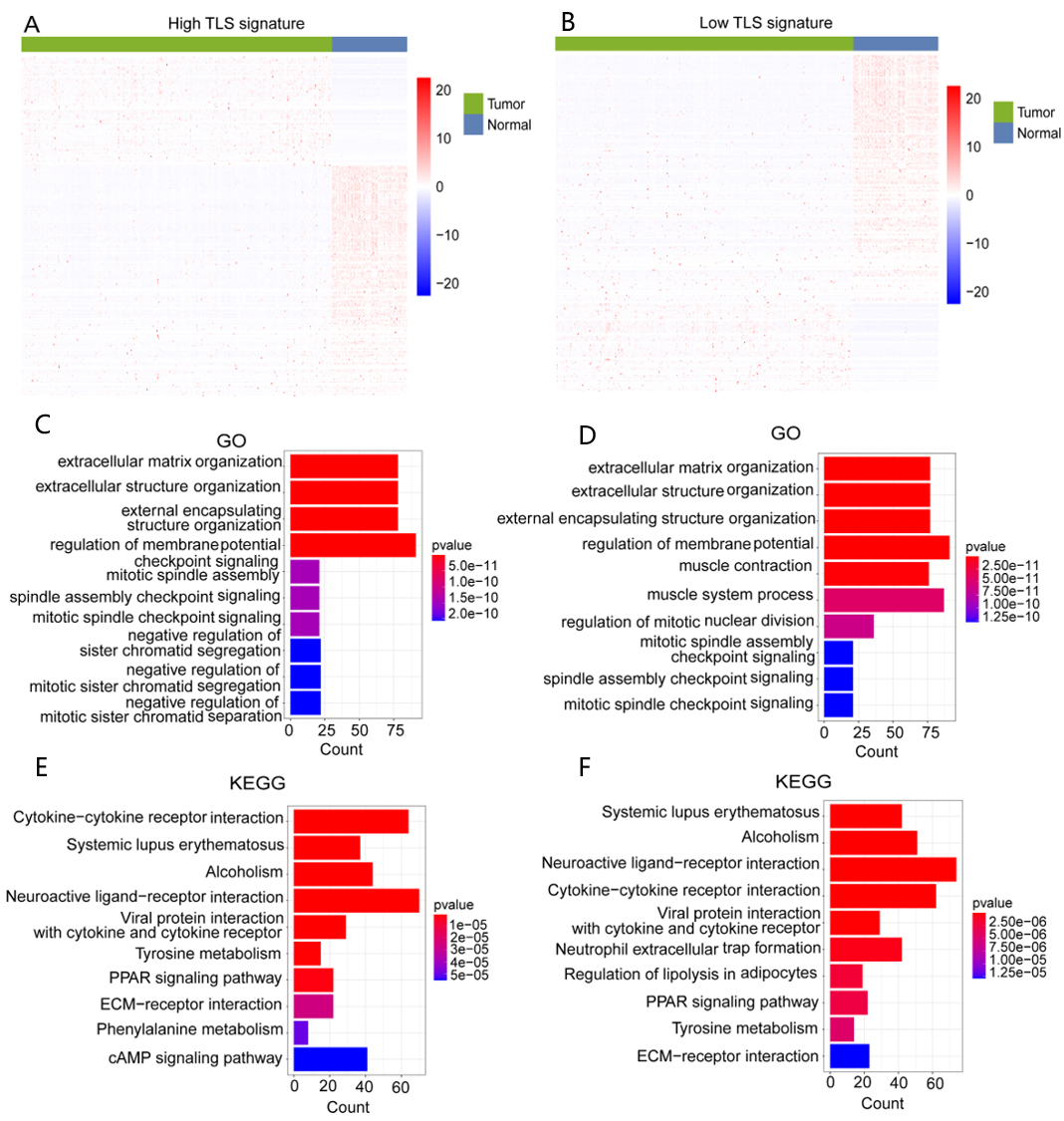


Figure S5 Differentially expressed genes and functions in different TLS signature subgroups in the TCGA-BRCA cohort. Heatmap displaying all differentially expressed genes (DEGs) in the high-TLS signature group (A) and low-TLS signature group (B). Gene Ontology (GO) enrichment analysis of the DEGs in the high-TLS signature group (C) and low-TLS signature group (D) (*p* < 0.05). Kyoto Encyclopedia of Genes and Genomes (KEGG) pathway analysis of the DEGs in the high-TLS signature group (E) and low-TLS signature group (F) (*p* < 0.05). The heatmap was plotted using the pheatmap package of R (version 1.0.12, URL: http://bioconductor.org/packages/release/bioc/vignettes/InteractiveComplexHeatmap/inst/doc/interactivate_indirect.html).


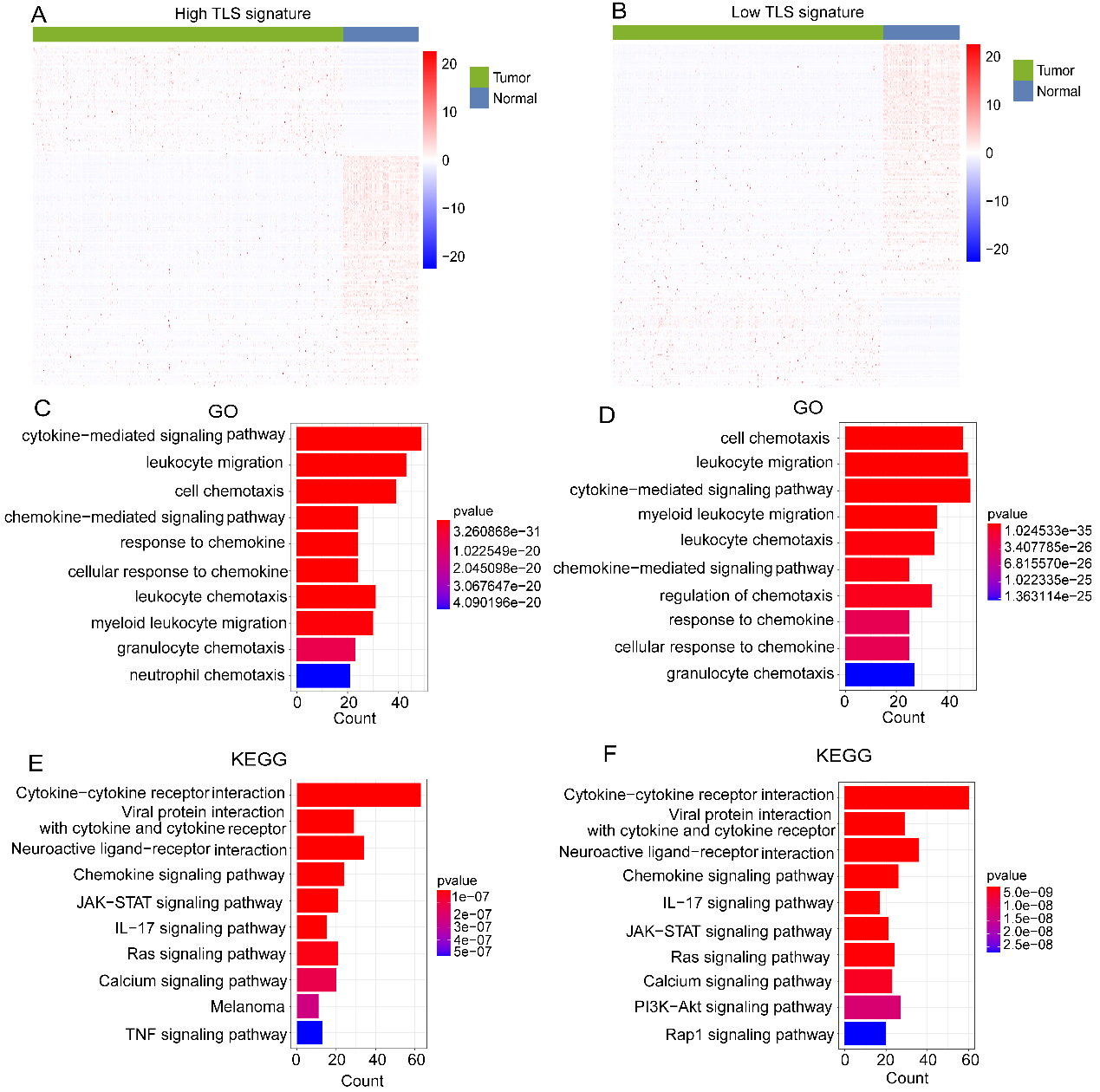


Figure S6 Differentially expressed immune-related genes and functions in different TLS signature subgroups in the TCGA-BRCA cohort. Heatmap displaying immune-related differentially expressed genes (DEGs) in the high-TLS signature group (A) and low-TLS signature group (B). Gene Ontology (GO) enrichment analysis of the immune-related DEGs in the high-TLS signature group (C) and low-TLS signature group (D) (*p* < 0.05). Kyoto Encyclopedia of Genes and Genomes (KEGG) pathway analysis of the immune-related DEGs in the high-TLS signature group (E) and low-TLS signature group (F) (*p* < 0.05). The heatmap was plotted using the pheatmap package of R (version 1.0.12, URL: http://bioconductor.org/packages/release/bioc/vignettes/InteractiveComplexHeatmap/inst/doc/interactivate_indirect.html).


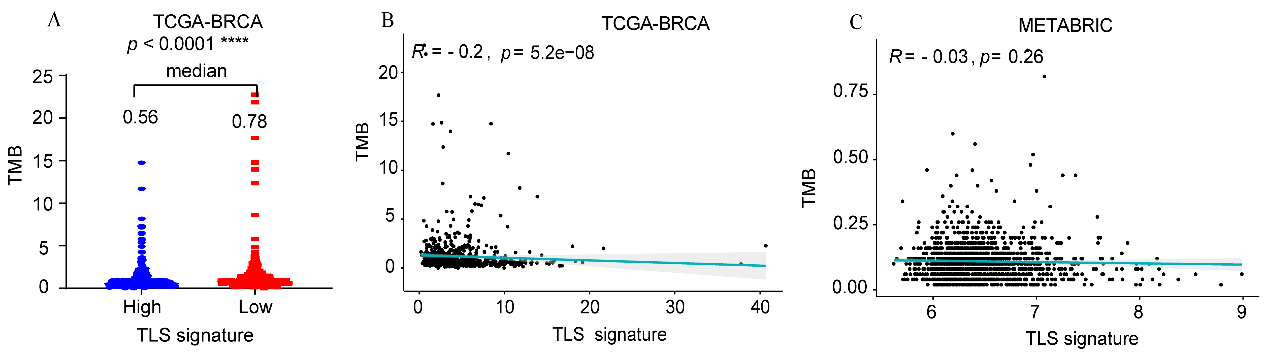


Figure S7 The relationship between TLS signature and total mutational burden (TMB). Differences in TMB between the TLS signature high and low groups in the TCGA- BRCA cohort (A). Correlation analysis between TLS signature and TMB in TCGA- BRCA (B) and METABRIC (C).
