# Supplementary material for: Genomic properties and clinical outcomes associated with tertiary lymphoid structures in patients with breast cancer
Source: Sci Rep. 2023 Aug 19;13:13542. doi: 10.1038/s41598-023-40042-7 (PMC10439954; doi:10.1038/s41598-023-40042-7)
Supplement: Supplementary file 3 — Supplementary Information 3. [file 41598_2023_40042_MOESM3_ESM.docx]

Table S1 Clinicopathological characteristics of the patients stratified by TLS signature

| **TCGA** | All (n%) | TLS signature low (n%) | TLS signature high (n%) | *p* value |
| --- | --- | --- | --- | --- |
| Total population | 866 (100) | 398 (46.0) | 468 (54.0) |  |
| Age |  |  |  | 0.897 |
| ≤50 years | 263 (30.4) | 120 (30.2) | 143 (30.6) |  |
| >50 years | 603 (69.6) | 278 (69.8) | 325 (69.4) |  |
| Menopausal status | |  |  | 0.577 |
| Pre-menopausal | 232 (26.8) | 103 (25.9) | 129 (27.6) |  |
| Post-menopausal | 634 (73.2) | 295 (74.1) | 339 (72.4) |  |
| T stage |  |  |  | **0.028** |
| T1 | 244 (28.2) | 97 (24.4) | 147 (31.4) |  |
| T2 | 490 (56.6) | 247 (62.1) | 243 (51.9) |  |
| T3 | 109 (12.6) | 45 (11.3) | 64 (13.7) |  |
| T4 | 23 (2.7) | 9 (2.3) | 14 (3.0) |  |
| N stage |  |  |  | 0.663 |
| N0 | 414 (47.8) | 189 (47.5) | 225 (48.1) |  |
| N1 | 290 (33.5) | 128 (32.2) | 162 (34.6) |  |
| N2 | 91 (10.5) | 47 (11.8) | 44 (9.4) |  |
| N3 | 66 (7.6) | 30 (7.5) | 36 (7.7) |  |
| Unknow | 5 (0.6) | 4 (1.0) | 1 (0.2) |  |
| TNM stage |  |  |  | **0.038** |
| I | 163 (18.8) | 62 (15.6) | 101 (21.6) |  |
| II | 492 (56.8) | 243 (61.1) | 249 (53.2) |  |
| III | 196 (22.6) | 84 (21.1) | 112 (23.9) |  |
| IV | 15 (1.7) | 9 (2.3) | 6 (1.3) |  |
| ER expression |  |  |  | **< 0.0001** |
| Yes | 678 (78.3) | 274 (68.8) | 404 (86.3) |  |
| No | 188 (21.7) | 124 (31.2) | 64 (13.7) |  |
| PR expression |  |  |  | **< 0.0001** |
| Yes | 588 (67.9) | 221 (55.5) | 367 (78.4) |  |
| No | 278 (32.1) | 177 (44.5) | 101 (21.6) |  |
| HER2 expression | |  |  | **0.022** |
| Yes | 165 (19.1) | 89 (22.4) | 76 (16.2) |  |
| No | 701 (80.9) | 309 (77.6) | 392 (83.8) |  |
| Death |  |  |  | **0.018** |
| Yes | 63 (7.3) | 38 (9.5) | 25 (5.3) |  |
| No | 803 (92.7) | 360 (90.5) | 443 (94.7) |  |

Abbreviations: TLS, tertiary lymphoid structure; TNM, tumor-node-metastasis; ER, estrogen receptor; PR, progesterone receptor; HER2, human epidermal growth factor receptor 2.

Table S2 Clinicopathological characteristics of the patients stratified by TLS signature

| **METABRIC** | All (n%) | TLS signature low (n%) | TLS signature high (n%) | *p* value |
| --- | --- | --- | --- | --- |
| Total population | 1399 (100) | 871 (62.3) | 528 (37.7) |  |
| Age |  |  |  | **0.001** |
| ≤50 years | 315 (22.5) | 170 (19.5) | 145 (27.5) |  |
| >50 years | 1084 (77.5) | 701 (80.5) | 383 (72.5) |  |
| Menopausal status | |  |  | **0.001** |
| Pre-menopausal | 315 (22.5) | 170 (19.5) | 145 (27.5) |  |
| Post-menopausal | 1084 (77.5) | 701 (80.5) | 383 (72.5) |  |
| T stage |  |  |  | **0.006** |
| T1 | 628 (44.9) | 364 (41.8) | 264 (50.0) |  |
| T2 | 708 (50.6) | 470 (54.0) | 238 (45.1) |  |
| T3 | 63 (4.5) | 37 (4.2) | 26 (4.9) |  |
| N stage |  |  |  | 0.461 |
| N0 | 752 (53.8) | 471 (54.1) | 281 (53.2) |  |
| N1 | 431 (30.8) | 275 (31.6) | 156 (29.5) |  |
| N2 | 145 (10.4) | 82 (9.4) | 63 (11.9) |  |
| N3 | 71 (5.1) | 43 (4.9) | 28 (5.3) |  |
| TNM stage |  |  |  | 0.100 |
| I | 475 (34.0) | 283 (32.5) | 192 (36.4) |  |
| II | 802 (57.3) | 518 (59.5) | 284 (53.8) |  |
| III | 113 (8.1) | 63 (7.2) | 50 (9.5) |  |
| IV | 9 (0.6) | 7 (0.8) | 2 (0.4) |  |
| ER expression |  |  |  | **< 0.0001** |
| Yes | 1080 (77.2) | 709 (81.4) | 371 (70.3) |  |
| No | 319 (22.8) | 162 (18.6) | 157 (29.7) |  |
| PR expression |  |  |  | **< 0.0001** |
| Yes | 738 (52.8) | 495 (56.8) | 243 (46.0) |  |
| No | 661 (47.2) | 376 (43.2) | 285 (54.0) |  |
| HER2 expression | |  |  | 0.063 |
| Yes | 172 (12.3) | 96 (11.0) | 76 (14.4) |  |
| No | 1227 (87.7) | 775 (89.0) | 452 (85.6) |  |
| Death |  |  |  | **< 0.0001** |
| Yes | 789 (56.4) | 526 (60.4) | 263 (49.8) |  |
| No | 610 (43.6) | 345 (39.6) | 265 (50.2) |  |

Abbreviations: TLS, tertiary lymphoid structure; TNM, tumor-node-metastasis; ER, estrogen receptor; PR, progesterone receptor; HER2, human epidermal growth factor receptor 2.

Table S3 Detailed R and p-value of correlation (spearman correlation) between TLS signature and other checkpoint members

| TCGA-BRCA | | | | METABRIC | | | |
| --- | --- | --- | --- | --- | --- | --- | --- |
| Gene_1 | Gene_2 | Correlation | *p*-value | Gene_1 | Gene_2 | Correlation | *p*-value |
| TLS | *PDL2* | 0.121 | 0.0002 | TLS | *PDL2* | 0.264 | 0.203 |
| TLS | *CD274* | 0.165 | 0.0009 | TLS | *CD274* | 0.23 | 0.057 |
| TLS | *CTLA4* | 0.164 | 0.0002 | TLS | *CTLA4* | 0.565 | 0.195 |
| TLS | *IDO1* | 0.095 | <0.0001 | TLS | *IDO1* | 0.461 | 0.659 |
| TLS | *LAG3* | 0.165 | 0.0025 | TLS | *LAG3* | 0.403 | 0.978 |
| TLS | *PDCD1* | 0.265 | 0.016 | TLS | *PDCD1* | 0.555 | 0.174 |
| TLS | *BTLA* | 0.267 | 0.022 | TLS | *BTLA* | 0.66 | 0.013 |
| TLS | *CD27* | 0.303 | 0.059 | TLS | *CD27* | 0.731 | 0.004 |
| TLS | *CD40* | 0.282 | 0.066 | TLS | *CD40* | 0.535 | 0.364 |
| TLS | *CD48* | 0.344 | 0.043 | TLS | *CD48* | 0.674 | 0.012 |
| TLS | *ICOS* | 0.189 | 0.0002 | TLS | *ICOS* | 0.584 | 0.166 |
